# Occurrence and Health-Risk Assessment of Trace Metals in Geothermal Springs within Soutpansberg, Limpopo Province, South Africa

**DOI:** 10.3390/ijerph17124438

**Published:** 2020-06-20

**Authors:** Olatunde Samod Durowoju, Georges-Ivo Ekosse Ekosse, John Ogony Odiyo

**Affiliations:** 1 Department of Hydrology and Water Resources, University of Venda, Private Bag X5050, Thohoyandou 0950, South Africa; john.odiyo@univen.ac.za; 2 Directorate of Research and Innovation, University of Venda, Private Bag X5050, Thohoyandou 0950, South Africa; Georges-Ivo.Ekosse@univen.ac.za

**Keywords:** geothermal springs, potential health risk, rock-water interaction, Soutpansberg, trace metals

## Abstract

Geothermal springs are natural geological phenomena that occur throughout the world. South Africa is blessed with several springs of this nature. Limpopo province contains 31% of all geothermal springs in the country. The springs are classified according to the residing mountain: Soutpansberg, Waterberg and Drakensberg. This study focused on the geothermal springs within the Soutpansberg region; that is, Mphephu, Siloam, Sagole and Tshipise. The study was aimed at assessing the occurrence and potential health risk associated with drinking water from geothermal springs within Soutpansberg. Geothermal springs and boreholes were sampled for a period of 12 months (May 2017–May 2018) to accommodate two major seasons in the study areas. The physicochemical and trace metal compositions of the geothermal springs and boreholes (tepid and hot) were analyzed using ion chromatography (IC) (Dionex Model DX 500) and inductively coupled plasma-mass spectrometer (ICP-MS). Trace metal concentrations of the geothermal springs and boreholes were within permissible drinking water guidelines by the South African National Standards (SANS) and World Health Organisation (WHO), with exception of mercury (Hg), which is high in summer season. The bioaccumulation from regular consumption could, however, result in negative effects. Pearson’s correlation revealed that there is a direct relationship between temperature and pH, and some of the trace metals (V, Zn, Hg, Pb). This implies dissolution of minerals (rock-water interaction) under slightly high temperature. Multivariate statistics further elucidate the relationship and possible sources of the trace metals. Therefore, it can be inferred that the rock-water interaction is the main geochemical process governing the release of trace metals in groundwater. Hazard Index values for both children and adults were higher than 1, and this implies that the communities are at high risk of non-cancer health effects. Further, As, Cr and Cd were found to be the highest contributors to the potential cancer risk in the study areas, with children having a higher risk than adults. Therefore, there is a need for clinical/epidemiological study, and regular monitoring and control measures, to verify actual prevalence of cancer and protect human health, particularly the children, within the study areas.

## 1. Introduction

Geothermal springs are natural geological phenomena which occur on all continents. They originate either from geologic platonic activity (volcanic origin), or from rainwater that percolates into the ground through permeable rocks or via conduits such as joints, faults and fracture zones in less permeable rocks (meteoric origin) [1]. In South Africa (SA), Limpopo Province has the highest number of geothermal springs. These springs are classified according to the dominating mountains; namely, Soutpansberg, Waterberg and Drakensberg [2]. There are 83 known geothermal springs in SA, out of which 24 are located in Limpopo Province [3,4]. This study focused on geothermal springs within Soutpansberg, namely Mphephu, Sagole, Siloam and Tshipise.

Geothermal springs are usually mineralized to a greater or lesser extent, depending on the characteristics of the geological formations associated with the circulating groundwater [5]. Odiyo and Makungo [6] reported that geochemical dissolution of rock increases with temperature, hence more mineralization of the geothermal springs. This also accounts for trace metals emanating from the geothermal springs to the surrounding soils and vegetation. People have used water from geothermal springs for different purposes for thousands of years [7]. Documentary and oral history reveal that geothermal springs were used for bathing, medicinal, religious, hygienic and social purposes across the world, for instance, India, Crete, Egypt, Turkey, Japan, Brazil and Canada [8,9].

Trace metals (TM) are also known as potentially toxic elements, heavy metals, micronutrients, and minor elements in the environment [10]. Heavy metals are natural components of the Earth’s crust. The natural occurrence of trace metals varies between rock types and certain bedrocks. These provide exceptionally high metal concentrations to overlying soils. Soils are of enormous environmental importance, being the media that support virtually all plant life, hence their potential for environmental pollution requires attention [11]. While soils are important receptacles for trace metals, they can also release them into the ecosystem. It is therefore important to understand the content, chemistry and geology of trace metals in geothermal water.

Studies have found that geothermal water may contain toxic elements such as arsenic, cadmium, chromium, selenium, and mercury [12,13,14], and radioactive elements such as uranium (U), thorium (Th) and Radon (Rn) [15,16]. However, the investigation of the impacts of trace metals from geothermal springs on the surface soil and vegetation is essential, since geothermal springs are rich in elements, owing to the rock-water interaction in the deep aquifer [17,18]. The situation is even more worrisome in South Africa, where geothermal springs are under-researched and under-utilized [7]. However, geothermal resources are gaining attention for their value even in South Africa, as predicted by Olivier et al. [7].

The use of geothermal spring water for domestic, recreational and agricultural purposes is prevalent in the study communities. Rural communities, such as those at Siloam village, produce most of their food on the land on which they live. When agricultural soils are contaminated, these trace metals are taken up by surrounding vegetation and consequently accumulate in their tissues [19]. Durowoju [20] showed that geothermal springs can enrich the surface soil with trace metals, which could possibly lead to contamination, particularly where the geothermal spring water is used for irrigation, recreation and agricultural purposes. This makes the community vulnerable to the effects of trace metals emanating from the geothermal springs to human beings via food chain [21]. Hence, there is a need to assess the potential health risk associated with trace metals in the geothermal springs.

Most of these geothermal springs are found in communities in which there is limited water availability (considered as reliable alternative source of water), particularly in developing countries such as Kenya, Ethiopia and South Africa, among others. The study areas (Mphephu, Sagole, Siloam and Tshipise) are rural settlements in Limpopo, South Africa, where people have little or no scientific knowledge of the effects of toxic contaminants from the geothermal springs on the ecosystem. Thus, spring water is used for various domestic purposes, swimming and irrigation as indicated earlier, with no clear understanding of the potential health effects of major and trace metals. Hence, the trace metal concentrations were used to assess the potential health risks in adults and children within selected communities (where the geothermal springs were located).

### Study Area

**Figure 1:**
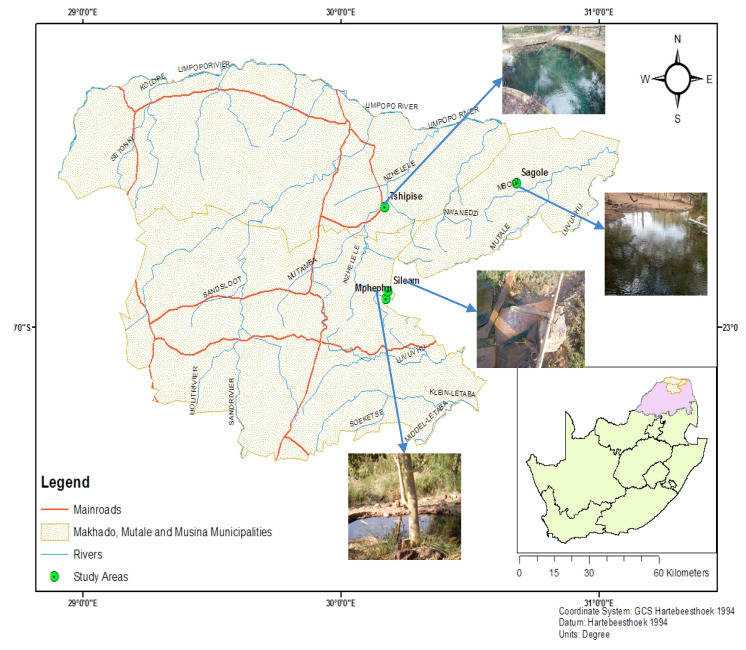
Study area map showing the geothermal springs within Soutpansberg.

The Mphephu and Siloam, Sagole, and Tshipise springs are located in Makhado, Mutale and Musina municipalities, respectively, in Vhembe District, Limpopo Province of South Africa (Figure 1). The study areas fall under quaternary catchments of the Nzhelele River catchment, which is located in the northern region of Limpopo Province, South Africa [22]. The study areas are characterized by great temperature variations in different seasons of the year, with temperatures in winter ranging from 16 °C to 22 °C, and in summer, from 22 °C to 40 °C [23]. The mean annual rainfall of Nzhelele ranges from 350 to 400 mm per annum [22]. More than 80% of the rainfall occurs in summer and only about 20% occurs in winter [24]. Brandl [25] reported that Tshipise and Siloam geothermal springs are underlain by intergranular and fractured aquifers, with borehole yields ranging between 0.1 L/s and 0.5 L/s. Sagole and Mphephu geothermal springs are underlain by fractured aquifers, with borehole yields ranging from 0.5 L/s to 2 L/s [26]. 

The study area is underlain by block-faulted Karoo Supergroup and Soutpansberg Supergroup rocks in the northern part of the Limpopo Province. These rocks have very low primary porosity, permeability and storage capacity, with limited groundwater flow [27]. Groundwater occurrence is mainly related to secondary hydrogeological features; that is, fault and joints, which present preferential pathways and thus enhance the potential for groundwater flow in the region. The geology determines the extent to which the reaction with the host rock proceeds, depending on the chemical composition of the rock and the rate at which water passes through the rock. Table 1 shows the surface geology and geological structures associated with geothermal springs within Soutpansberg.

## 2. Materials and Methods 

### 2.1. Sampling and Sample Pretreatment

Geothermal spring water samples were collected from Mphephu, Sagole and Tshipise springs. At Siloam, the geothermal spring was sampled for a season because it dried up, and is still dry to date. However, two different boreholes within Siloam village with similar thermal properties to a geothermal spring were explored. These boreholes were sampled following standard groundwater sampling procedure [29]. Sampling in all the springs and thermal boreholes was carried out for a period of 12 months (May 2017–April 2018) to accommodate two major seasons in the study areas. It was done once a month (thrice per season), specifically winter and summer seasons, to establish the seasonal effect on the parameters [20].

Representative samples were obtained through random sampling, in which water was sampled from every part of the spring, where possible with a plastic cup as recommended by Harvey [30]. The plastic containers were rinsed properly with the spring water to avoid cross-contamination. The samples were kept inside the cooler box and finally stored in the refrigerator at 4 °C. All the water samples were collected in 2 L plastic containers before transporting them to the laboratory for sample pre-treatment. The water samples were not filtered, because there is need to analyze the water in its original status, but acidified with concentrated HNO_3_ to pH < 2 (normally, 2 mL of concentrated acid per liter) following United State Environmental Protection Agency (USEPA) [29]. Parameters such as temperature, pH, electrical conductivity (EC), total dissolved solids (TDS) and alkalinity were measured in triplicate in situ, and the mean results are presented. The water samples codes are SGW and SGS; TSW and TSS; MPW and MPS in winter and summer, for Sagole, Tshipise and Mphephu geothermal springs, respectively. Whereas at Siloam village, SAW for geothermal springs, SH1 and SH2 for thermal boreholes, BH1 and BH2 for tepid boreholes, and SCC for community treated tap water are the sampling codes. TTP represents treated water from municipality at Tshipise.

The measurements of the pH, temperature, EC and TDS of the water samples were carried out in situ using Multimeter (Multi 340i/SET, Expotech, Houston, TX, USA) and at the laboratory [Agricultural Research Council (ARC) in Pretoria, South Africa]. 

The sampling and pretreatment were carried out using standard procedures, and samples were preserved properly for further chemical analyses. Quality assurance/quality control of field samples was carried out for geothermal spring water sampling to enhance sampling integrity, increase the confidence of analytical data, and prevent reporting positives caused by contamination.

### 2.2. Experimental Analyses

The geothermal spring and borehole samples were analyzed for trace metals using Inductively coupled plasma mass spectrometry (ICP-MS) with a dilution factor of 10. All the measurements were carried out in triplicate to obtain a mean value. Trace metals were analyzed using ICP-MS after the background check-up of the equipment (calibration). The method detection limit (MDL) for each trace metal was obtained by U.S. EPA method 200.8 [31]. In order to validate the analytical methodology, recovery studies were performed. Known concentrations of the test analyte were added to the sample. The concentrations of both the spiked and unspiked samples were determined and percentage recovery was obtained. The validation test performed on the analytical methods employed gave reproducible results with acceptable recoveries. Recovery percentages were 95.9% for Cr, 97.6% for Co, 96.3% for Ni, 91.2% for Cu, 94.7% for Zn, 93.6% for As, 93.8% for Cd, 97.2% for Ba, 92.6% for Be, 94.3% for Mn, 91.5% for Sb, 98.1% for Se, 96.2% for V and 96.2% for Pb. The MDL for each trace metal was 0.01 µg/L (As), 0.04 µg/L (Ba), 0.03 µg/L (Be), 0.03 µg/L (Cd), 0.08 µg/L (Cr), 0.003 µg/L (Co), 0.01 µg/L (Cu), 0.2 µg/L (Hg), 0.04 µg/L (Mn), 0.03 µg/L (Ni), 0.02 µg/L (Pb), 0.02 µg/L (Sb), 0.5 µg/L (Se), 0.05 µg/L (V) and 0.2 µg/L (Zn).

### 2.3. Assessment of Health Risk from Geothermal Springs

Common exposure pathways for water are the dermal absorption and ingestion routes (Table 2) [32,33]. Hence, exposure dose—to assess the human health risk—was calculated using the following equations, as adapted from the U.S. EPA risk assessment guidance for Superfund (RAGS) methodology [32,33].

(1) $${\mathrm{Exp}}_{\mathrm{ingestion}}=\frac{C_{water}\times IR\times EF\times ED}{BW\times AT}$$

(2) $${\mathrm{Exp}}_{\mathrm{dermal}}=\frac{C_{water}\times SA\times KP\times ET\times EF\times ED\times CF}{BW\times AT}$$

where, Exp_ingestion_: exposure dose through ingestion of water (mg/kg/day); Exp_dermal_: exposure dose through dermal absorption (mg/kg/day); *C_water_*: average concentration of the estimated trace metals in water (μg/L); Kp: dermal permeability coefficient in water, (cm/h)—0.001 for Cu, Mn, Fe and Cd, while 0.0006 for Zn, 0.002 for Cr and 0.004 for Pb.

Potential non-carcinogenic risks due to exposure of trace metals were determined by comparing the calculated contaminant exposures from each exposure route (ingestion and dermal) with the reference dose (RfD) (Table 2), using Equation (3), to generate hazard quotient (HQ) toxicity potential of an individual via the two pathways using Equation (4) (hazard index).

(3) $$\mathrm{HQ}=\frac{Exp}{Rfd}$$

(4) $$\mathrm{HI}=\sum_{i=1}^{n}HQ$$

Chronic daily intake (CDI) of trace metals through ingestion was calculated using Equation (5):

(5) $$\mathrm{CDI}=C_{water}\times \frac{IR}{BW}$$

where *C_water_*, IR and BW represent the concentrations of the trace metals in water, average daily intake of water and body weight, respectively. Carcinogenic risk (CR) through ingestion pathway was estimated using Equation (6).

(6) $$\mathrm{CR}=\frac{Exp}{exP}$$

where *exP* is the carcinogenic slope factor, and is represented in Table 3.

### 2.4. Data Analyses

All values from chemical analyses were presented as mean values in tables and figures. Multivariate statistics, such as principal component analysis (PCA)/factor analysis (FA), and hierarchical agglomerative analysis (HAC), were performed using XLSTAT (Addinsoft Inc. New York, NY, USA) statistical software [38]. The PCA was used to establish major variations and relationships among the different trace metals and physicochemical parameters. HAC dendrogram shows the degree of similarity/dissimilarity amongst parameters obtained at different sites within Soutpansberg. Pearson correlation analysis was carried out to determine the relationship among the parameters. The calculation of risk indices was done by Microsoft Office Excel, version 2018(Microsoft Corporation, WA, USA).

## 3. Results and Discussion

### 3.1. Occurrences and Distributions of Trace Metals in Geothermal Springs and Boreholes

Table 4 shows the mean values for trace metal concentrations and physicochemical parameters in the geothermal springs, hot boreholes and tepid boreholes. Results show that geothermal springs are highly mineralized owing to their geological formations, as supported by Todd [5]. More mineralization of the geothermal springs was aided by the thermal gradient (temperature) leading to more mineral dissolution in water [6,39]. Similar studies by Leal-Acosta et al. [39] and Rezaei et al. [40] reported highly mineralized water, with toxic metals such as Hg and As, among others, as present in geothermal systems, which could impact on their surroundings. A previous study by Durowoju et al. [17] has shown that the geothermal spring has potential for enriching the surrounding soils with trace metals, which are absorbed by surrounding vegetation.

The obtained values were compared with the standard guidelines for drinking water by SANS [41] and WHO [42]. The physicochemical parameters of the studied geothermal springs were within the recommended guidelines, except for pH at Siloam (SAW and SH2). pH values ranged from 7.17 to 9.39, which implies that the waters are alkaline in nature. This could be a result of the water types’ Na-HCO_3_ and NaCl from the studied geothermal springs, as reported by Durowoju et al. [43]. The water temperature of springs ranges between 41.3 °C and 68.9 °C (Table 4), and are classified as: Mphephu (MPS and MPW) and Sagole (SGS and SGW) springs, and Siloam (SH1 and SH2) boreholes, are thermal (hot) water with temperatures ranging between 41 °C to 49 °C; Siloam (SAW) and Tshipise (TSS and TSW) geothermal springs can be classified as scalding (hyperthermal) with temperature ranging between 53 °C and 69 °C. The seasonal variation leads to the fluctuation of the thermal property of the springs. During summer, there is high rainfall and more underground water (coupled with high flow rate), which is heated by the geothermal gradient of 2−3 °C per 100 m [44]. This implies that geothermal spring water with high temperature emanates from a deeper source.

Generally, the trace metal concentrations of the geothermal springs and boreholes within Soutpansberg were within the drinking water permissible guidelines by the SANS and WHO, except for mercury (Hg), which is high in summer (>1 μg/L). This high mercury concentration could be associated with igneous activity and circulating geothermal fluids that precipitate around mineral springs, geysers and fumaroles, particularly during summer, when there is high rainfall [39,45]. Though trace metal concentrations were within the drinking water guidelines, the accumulation in the human body could result in adverse effects, considering that some of these metals are carcinogenic in nature.

**Figure 2:**
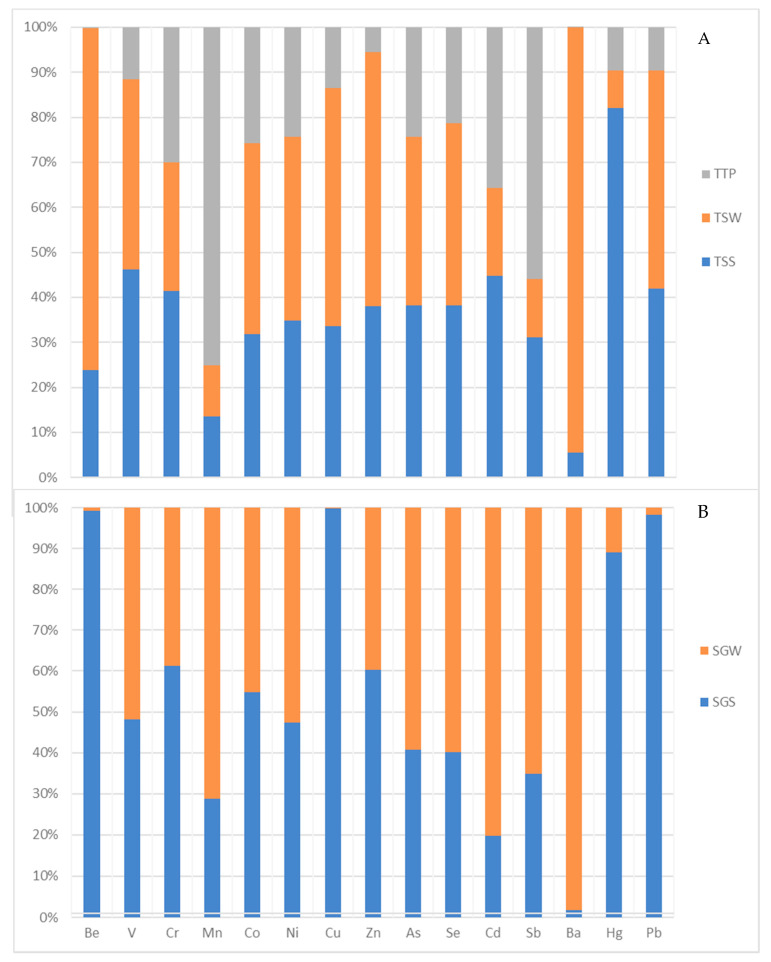
Variations of trace metals concentrations in (**A**) Tshipise geothermal spring and tepid borehole, and (**B**) Sagole geothermal spring.

**Figure 3:**
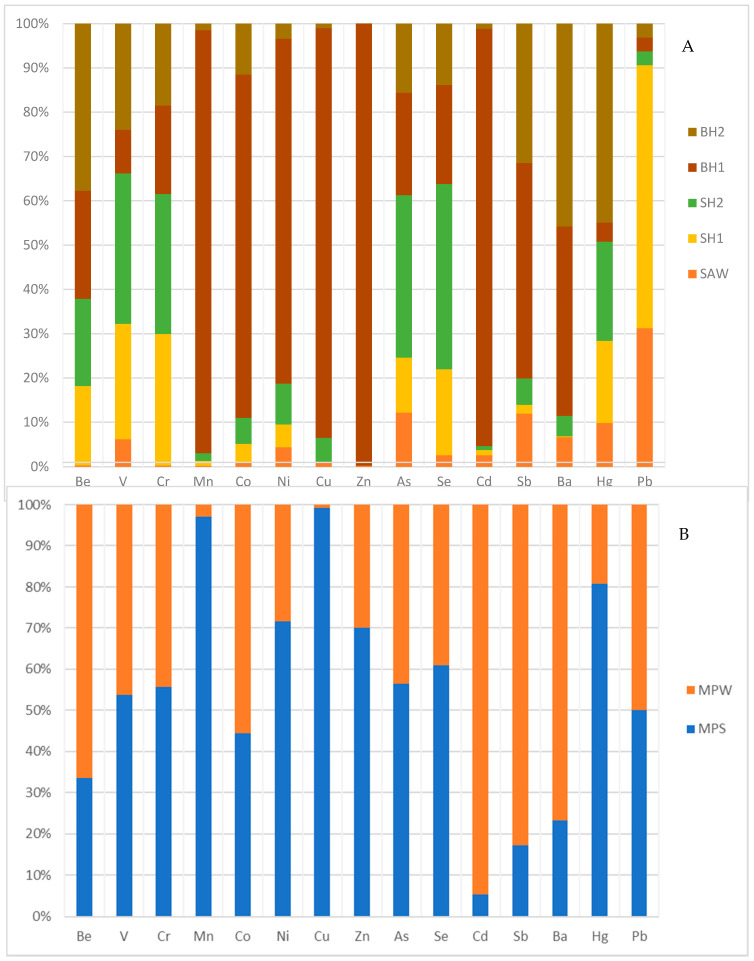
Variations of trace metals concentrations in (**A**) Siloam geothermal spring, hot and tepid boreholes, and (**B**) Mphephu geothermal spring.

Generally, the mean trace metal concentrations were higher in summer compared to winter, except for some trace metals, such as Be, Co, Ni, Cu, Zn, Se, Ba, at different sites with anomalous concentrations. This could be attributed to the temperature differences and more rainfall, leading to more dissolution of the host rock (minerals) in summer (Figure 2 and Figure 3). Figure 2 and Figure 3 show clearly the variations in the trace metal concentrations in the geothermal springs and boreholes (hot and tepid). In all the sites, there is approximately 1 °C difference in the thermal property of the geothermal spring in summer compared to winter. These high temperatures in summer result in more transfer of moisture (evaporation and evapotranspiration) to the atmosphere until it reaches the dew point—hence there is potential for more intense rain during this period—and contribute to more dissolution of the host rock as explained above.

The mean trace metals concentrations within the study areas were in relatively good agreement during summer for geothermal springs (Table 4). As stated earlier, more rainfall in summer (wet) enhances the rock-water interaction at the deep aquifer of the geothermal spring, and more trace metals are released into the water body at the surface. Therefore, there are more trace metals in the geothermal spring water during summer (wet) than in the winter (dry). At Siloam, an anomalous trend was found among the geothermal spring, hot borehole and tepid boreholes, where the boreholes were in some cases more enriched with trace metals than the geothermal spring. This could possibly be linked to the geology of the area, although the geology of an area is complex and differs from one point to another [4,7]. For instance, two homesteads where the borehole water was sampled are next to one another, and their water characteristics are different (one is hot and the other is cold). The chemical and isotopic parameters of these boreholes differ, as reported by Durowoju et al. [43]. Hence, there is a possibility of common host rock and minor faults connecting the aquifer of geothermal spring and boreholes, thus there is aquifer interconnectivity [43].

Relationships of trace metals in water with some physicochemical parameters were evaluated using the Pearson’s correlation (Table 5). There is a direct relationship between temperature and alkalinity, pH, EC, V, Zn, Hg and Pb. This means that an increase in water temperature results in increases in EC, pH (leading to high alkalinity), and trace metals such as V, Zn, Hg and Pb. This is an indication of dissolution of minerals (rock-water interaction) under high temperature. Furthermore, there was a negative correlation between temperature and other trace metals (Be, Cr, Mn, Co, Ni, Cu, As, Se, Cd, Sb, Ba). This means that these trace metals are in a good relationship with one another, or perhaps have some common minerals in their compositions. This study revealed that pH has a negative correlation with all the trace metals (Be, Cr, Mn, Co, Ni, Cu, As, Se, Cd, Sb, Ba, V, Zn and Hg) except Pb. This means that increases in pH (basic) result in decreases in trace metal concentrations in the geothermal springs and boreholes, resulting in less indication of trace metals pollution (insoluble) [46]. This is in support of a previous study that stated that most metals seem to be more toxic in an acidic state [47]. The conductivity values had a significant positive relationship with all trace metals, such as Be, V, Mn, Co, Ni, Cu, Zn, Cd, Sb, Ba, Hg and Pb, except the high-alkaline Cr, As and Se. It could be inferred that the changes of physicochemical parameters depend on how seasons affect the levels of some metals [48]. 

**Figure 4:**
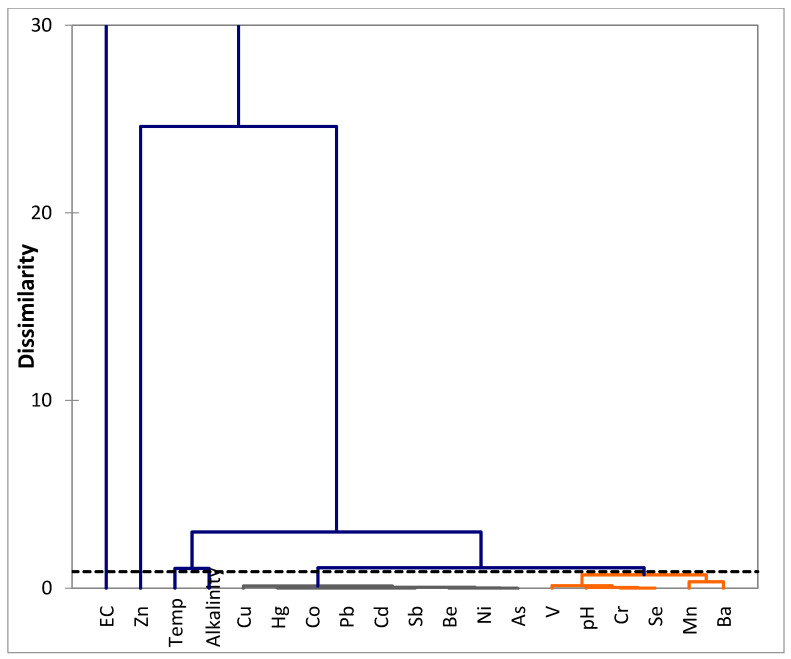
Dendrogram showing the spatial clustering of trace metals in geothermal spring/borehole water samples, based on the hierarchical cluster analysis using Ward’s method.

The relationships among the trace metals were further determined by hierarchical cluster analysis (HCA) using XLSTAT statistical software [38]. They were grouped into clusters based on the similarities and dissimilarities between different metals (Figure 4). Dendrogram analysis produced six clusters for the spatial distribution of trace metals of the samples; clusters 2 and 5 include pH and all the trace metals, except Cu. These metals are likely present in the geothermal springs and boreholes due to agricultural run-off or atmospheric deposition in the study areas [49]. This is corroborated by the findings from the Pearson correlation matrix, which indicates trace metals are insoluble at higher pH (basic medium), hence the negative correlation. Clusters 1, 3, 4 and 6 are temperature, conductivity, alkalinity and Cu, respectively, occurring independently. The results of cluster analysis support the correlation results, which suggest that the selected metals are from anthropogenic and natural sources.

**Figure 5:**
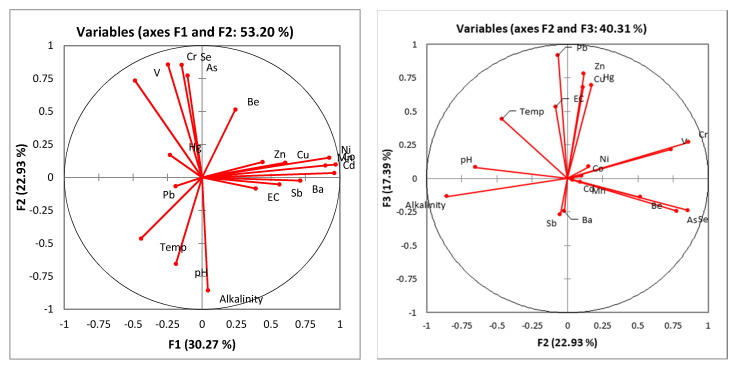
The principal component analysis (PCA) biplots showing the relationships between trace metals in the geothermal spring and borehole samples.

The PCA/FA loading factors for the trace metals in the geothermal spring and borehole samples, taken within Soutpansberg, are shown in Table 6. For both seasons (winter and summer), five important principal components (PCs) were significant, with eigenvalues > 1, explaining higher total variance of 30.27%, 53.20%, 70.59%, 79.00% and 86.61%, respectively (Table 6 and Figure 5). The factor loadings show that F1 (30.27%) has high loadings of Mn, Co, Ni, Cu, Cd, Sb and Ba; F2 (22.93%) has high loadings of Be, V, Cr, As and Se; F3 (17.39%) has high loadings of EC, Cu, Zn, Hg and Pb; F4 (8.42%) has the highest loading of Be; and F5 (7.60%) has high loading of pH. Multivariate analysis using PCA/FA is very useful as a monitoring tool, to identify the multiple sources of contaminants and relationships with trace metals in groundwater (Figure 5). The five factors are interrelated and are indicative of rock-water interactions, such as thermal gradient, mineral dissolution and ion exchange, as the major geochemical processes governing the groundwater chemistry. This supports the previous findings in water types [43], and confirms that the rock-water interaction is one of the major processes controlling the chemistry of the geothermal springs and boreholes.

### 3.2. Evaluation of Potential Human Health Risk Associated with Trace Metals in Geothermal Springs and Boreholes

The levels of exposure through ingestion and dermal contact were estimated, since these are the major exposure pathways of geothermal springs and boreholes in the communities. Possible health risk associated with exposure through ingestion depends on the weight, age and volume of groundwater consumed by an individual (children and adults), as presented in Table 7. In most of the study areas, the children’s chronic daily intake was higher than the adults, indicating that children are more susceptible to potential health risk associated with the consumption of trace metals in groundwater. When the hazard index (HI) is less than 1, there is no obvious risk to the population, but if these values exceed 1, there may be concern for potential non-carcinogenic effects [33,50]. The calculated cumulative hazard quotients (HQ) for all the trace metals served as a conservative assessment tool, to estimate high-end risk rather than low end-risk, to protect the public. Calculated HI served as a screening value to determine whether there is a major significant health risk that exposure to trace metals in the groundwater may pose to the community, and if there is any difference in total health risk during the study period. For the adult population, the calculated values for HI were less than 1 in dermal intakes. That said, calculated HI (summation of the HQs) for all the exposure pathways was 1.23, a value greater than 1 that is due to the ingestion pathways. Trace metals such as Be, Cr, Hg and As are the main contributors (HI values range from 0.1 to 0.5), hence, the adult population was at risk of non-carcinogenic diseases.

For children, calculated HI (summation of the HIs) was 54.7, with Be, Se, As, Mn, Cr, Hg and V being the major contributors (HI values range from 1.04 to 9.94), through the ingestion pathway. This high value indicated trace metal pollution, that may pose a very high non-cancer health risk to children living in those communities. In general, the health risk assessment index, using the overall non-carcinogenic risk assessment (HI), CDI and HQ via ingestion and dermal adsorption routes, was greater than 1. This is an indication that groundwater poses more significant health threats, to both adults and children, via the pathways [33,50], however, measures should be made to avoid accumulation of trace metals that pose adverse health problems, especially in children (Appendix A). According to the World Health Organization [40] report, children are a population vulnerable to health risks, because they drink more water, consume more food, and breathe more air in proportion to their weight. Children’s immune, digestive, reproductive and nervous systems are still growing and at early development, and thus exposure to toxic elements causes irreversible damage. 

The carcinogenic risks of only Cr, Cd, As and Pb were calculated for both adults and children, because the values of carcinogenic slope factors for the other trace metals could not be found in the literature (Table 6). According to regulatory bodies, the acceptable carcinogenic risk values range between 10^−6^ and 10^−4^ for an individual (children and adults) [37,51]. In this study, Cr, Cd and As were found to be the highest contributors to the cancer risk in adults and children (Table 8). Pb poses potential carcinogenic risks to children in all the sites in both seasons, and this is of great concern and requires attention. Conversely, Cd also poses potential cancer risk in children at all sites, but falls within the acceptable limits for an adult population, except for in MPW and BH1. Hence, Cd poses a cancer risk in adults at MPW and BH1. SAW (Siloam), having 4.04 × 10^−5^ in Cr in the adult population, does not pose a cancer risk to this population, unlike other sites. It is also interesting to note that both populations (adults and children) in all sites show higher risk in summer compared to winter season. This is in accordance with higher trace metal concentration occurrence in summer compared to the winter season. Furthermore, the treated water (SCC and TTP) were within the recommended guidelines, and hence there is no cancer risk in the consumption of such water. Therefore, proper monitoring and control measures (civic education, routine sampling and remediation, among others) to protect human health within the study areas should be implemented for safety by relevant stakeholders.

## 4. Conclusions

The trace metal concentrations in the geothermal springs and boreholes are within drinking permissible guidelines established by SABS and WHO, with the exception of mercury (Hg), which is high in summer. Presence of Hg could be associated with igneous activity and circulating geothermal fluids, which precipitate around geothermal springs, geysers and fumaroles, especially during summer when there is high rainfall. Pearson’s correlation revealed that there is a strong relationship between the temperature and pH, which both correlate with some of the trace metals. This is an indication of dissolution of minerals (rock-water interaction) under slightly high temperatures. HCA and PCA/FA further elucidated these relationships and the possible sources of the trace metals. It can be inferred that the rock-water interaction is the main geochemical process governing the formation of trace metals in groundwater. 

Even though trace metal concentrations were within the drinking water guideline, their accumulations in the human body could result in adverse effects, considering that some of these metals are carcinogenic in nature. HI values for both children and adults were higher than 1, and this implies that the communities have a high risk of non-cancer health conditions. The ingestion pathway is the major pathway, with trace metals such as As, Be, Se, Cr, Co, Mn, Hg, V and Zn as the main drivers. As, Cr and Cd were found to be the highest contributors to the cancer risk in study areas, with children (1 in every 10 children) having a higher risk than adults (1 in every 1000 adults). Further, there is higher potential health risk in consuming the geothermal springs in the summer compared to the winter season. Therefore, there is a need for clinical/epidemiological studies and regular monitoring and control measures, to verify the actual prevalence of cancer and protect human health, particularly the children’s, within the study areas.

## Figures and Tables

**Table 1:** Geology and geological structures associated with geothermal springs.

Sampling Site	Surface Geology and Geological Structures
Mphephu	Quartzite and Sandstone, Reverse fault between Waterberg Group quartzite and Dominion Reef lava
Sagole	Mudstone, shale, subordinate micaceous sandstone, carbonaceous shale, siltstone, micaceous sandstone. Mikambeni Formation and Madzaringwe Formation, Karoo Supergroup
Siloam	Basalt, minor tuff, Sibasa Formation, Soutpansberg Group
Tshipise	Basalt, minor andesite, cream-coloured sandstone, dolerite sills and dykes Intersection of 2 post-Permian faults in upper Karoo

According to the Geological Survey: 1:250,000 Messina; Kent [3,28].

**Table 2:** Parameters used for the health risk assessment through different exposure pathways of water [33,34,35].

Parameter	Unit	Child	Adult
Body weight (*BW*)	Kg	15	70
Exposure frequency (*EF*)	days/year	365	365
Exposure duration (*ED*)	Years	6	70
Ingestion rate (*IR*)	L/day	1.8	2.2
Skin surface area (*SA*)	cm^2^	6,600	18,000
Particulate emission factor (*PEF*)	m^3^/kg	1.3 × 10^9^	1.3 × 10^9^
Exposure time (ET)	h/day	1	0.58
Averaging Time (AT)	Days	365 × 6	365 × 70
Conversation factor (CF)	L/cm^3^	0.001	0.001

**Table 3:** Reference doses (RfD) in mg/kg/day, and Cancer Slope Factors (exP) for the different trace metals [32,36,37].

TM	*RfD_ingestion_*	*RfD_dermal_*	*exP*
As	3.00 × 10^−4^	3.00 × 10^−4^	1.50
Ba	2.00 × 10^−1^	-	-
Be	2.00 × 10^−4^	-	-
Cd	1.00 × 10^−3^	1.00 × 10^−3^	6.30
Cr	3.00 × 10^−3^	3.00 × 10^−3^	5.00 × 10^−1^
Co	2.00 × 10^−2^	5.70 × 10^−6^	-
Cu	3.70 × 10^−2^	2.40 × 10^−2^	-
Hg	3.00 × 10^−4^	3.00 × 10^−4^	-
Mn	2.40 × 10^−2^	1.43 × 10^−3^	-
Ni	2.00 × 10^−2^	5.60 × 10^−3^	-
Pb	3.50 × 10^−3^	5.25 × 10^−4^	8.50 × 10^−3^
Sb	4.00 × 10^−4^	-	−
Se	5.00 × 10^−3^	-	-
V	5.04 × 10^−3^	-	-
Zn	3.00 × 10^−1^	7.50 × 10^−2^	-

**Table 4:** Mean physicochemical parameters and trace metal concentrations of the geothermal springs and boreholes within Soutpansberg.

	SANS; WHO	TSS	TSW	SGS	SGW	MPS	MPW	SAW	SH1	SH2	BH1	BH2	SCC	TTP
Temp (°C)		55.40	54.60	44.80	42.40	42.70	41.30	67.70	45.20	48.40	22.40	21.40	20.10	22.50
pH	6−9	8.47	8.46	7.98	8.82	8.15	8.05	**9.39**	8.86	**9.19**	8.17	8.1	7.17	8.17
EC (µS/cm)	˂750	745.00	746.67	347.33	330.00	365.00	335.00	340.00	630.00	330.00	690.00	730.00	90.00	290.00
Alkalinity (mg/L)		10.75	11.12	6.5	10.50	6.00	12.5	107.52	10.00	12.00	25.50	17.50	2.00	11.50
Be (μg/ L)		1.83	5.84	1.34	0.01	2.60	5.13	0.05	3.21	3.53	4.37	6.76	5.06	0.01
V (μg/ L)		18.36	16.74	13.51	14.59	16.28	13.96	3.21	13.54	17.63	5.12	12.46	17.83	4.62
Cr (μg/ L)	50; 100	12.46	8.64	10.48	6.64	10.57	8.40	0.09	10.40	11.08	6.99	6.48	12.14	9.03
Mn (μg/ L)	500; 1000	2.67	2.22	10.30	25.55	36.60	1.06	0.24	1.25	1.95	107.50	1.66	1.52	14.69
Co (μg/ L)		0.21	0.28	0.43	0.36	0.29	0.36	0.04	0.19	0.26	3.42	0.51	0.24	0.17
Ni (μg/ L)	20; 150	2.25	2.64	0.99	1.11	2.14	0.84	0.71	0.82	1.48	12.52	0.55	1.88	1.57
Cu (μg/ L)	2000; 1000	11.97	18.75	30.58	0.06	1.28	0.01	0.35	0.01	1.84	31.39	0.34	2.15	4.82
Zn (μg/ L)	3000; 5000	312.90	464.85	294.38	194.59	49.35	21.00	0.95	0.01	0.01	350.90	0.01	0.01	44.86
As (μg/ L)	10; 10	2.04	2.01	1.35	1.97	2.72	2.10	1.01	1.03	3.04	1.92	1.29	3.05	1.30
Se (μg/ L)		5.83	6.18	3.86	5.74	10.02	6.42	0.68	5.07	10.95	5.85	3.62	10.94	3.25
Cd (μg/ L)		0.06	0.02	0.01	0.05	0.01	0.14	0.02	0.01	0.01	0.73	0.01	0.01	0.05
Sb (μg/ L)	5; 5	0.05	0.02	0.03	0.06	0.04	0.17	0.03	0.01	0.02	0.12	0.08	0.01	0.10
Ba (μg/ L)		1.54	26.39	0.78	42.39	8.79	29.00	10.42	0.38	7.20	67.32	71.98	6.84	0.01
Hg (μg/ L)	1; 1	**6.11**	0.62	**3.26**	0.40	**1.82**	0.43	0.35	0.66	0.80	0.15	**1.60**	0.46	0.72
Pb (μg/ L)	10; 20	0.28	0.33	0.49	0.01	0.01	0.01	0.09	0.17	0.01	0.01	0.01	0.01	0.06

SGW and SGS, TSW and TSS, and MPW and MPS in winter and summer, for Sagole, Tshipise and Mphephu geothermal springs, respectively. At Siloam village, SAW—geothermal springs; SH1 and SH2—thermal boreholes; BH1 and BH2—tepid boreholes; and SCC—community treated tap. TTP—treated municipality water at Tshipise. Values in bold indicate exceedance of SANS [39] and WHO [40] guideline values for drinking water.

**Table 5:** Pearson correlation matrix showing the relationships between trace metals and physicochemical parameters in geothermal spring and borehole water.

Variables	Temp	pH	EC	Alk	Be	V	Cr	Mn	Co	Ni	Cu	Zn	As	Se	Cd	Sb	Ba	Hg	Pb
Temp	1.00																		
pH	**0.71**	1.00																	
EC	0.13	0.21	1.00																
Alkalinity	0.48	**0.55**	−0.04	1.00															
Be	−0.36	−0.42	0.37	−0.33	1.00														
V	0.13	−0.22	0.02	**−0.65**	0.37	1.00													
Cr	−0.25	−0.48	−0.04	**−0.86**	0.22	**0.72**	1.00												
Mn	−0.38	−0.13	0.20	−0.03	0.00	−0.40	−0.12	1.00											
Co	−0.42	−0.17	0.34	0.00	0.23	−0.39	−0.13	**0.93**	1.00										
Ni	−0.34	−0.16	0.34	0.00	0.19	−0.34	−0.05	**0.92**	**0.96**	1.00									
Cu	−0.06	−0.23	0.35	−0.12	0.05	−0.15	0.12	0.54	**0.63**	**0.64**	1.00								
Zn	0.18	−0.06	0.53	−0.18	0.03	0.10	0.11	0.38	0.41	0.49	**0.81**	1.00							
As	−0.14	−0.30	−0.35	−0.44	0.30	**0.64**	0.54	0.08	0.02	0.12	−0.11	−0.03	1.00						
Se	−0.21	−0.32	−0.29	**−0.58**	0.37	**0.70**	**0.68**	0.06	0.00	0.09	−0.15	−0.11	**0.95**	1.00					
Cd	−0.35	−0.12	0.30	0.06	0.17	−0.44	−0.17	**0.90**	**0.98**	**0.96**	**0.58**	0.39	0.00	−0.04	1.00				
Sb	−0.36	−0.24	0.09	−0.04	0.15	−0.37	−0.21	0.37	0.44	0.34	0.09	0.03	−0.13	−0.22	0.54	1.00			
Ba	−0.42	−0.10	0.43	0.03	0.48	−0.22	−0.41	0.51	**0.62**	0.48	0.16	0.19	−0.09	−0.15	**0.57**	0.51	1.00		
Hg	0.26	−0.09	0.33	−0.21	−0.18	0.38	0.44	−0.19	−0.20	−0.15	0.26	0.33	−0.02	−0.05	−0.21	−0.13	−0.30	1.00	
Pb	0.43	0.00	0.30	−0.10	−0.15	0.19	0.23	−0.26	−0.19	−0.15	**0.60**	**0.61**	−0.34	−0.31	−0.22	−0.34	−0.39	**0.56**	1.00

Values in bold are different from 0 with a significance level alpha = 0.05.

**Table 6:** Factor loadings of the trace metals concentrations and some physicochemical parameters.

	F1	F2	F3	F4	F5
Temperature	−0.4432	−0.4625	0.4421	−0.2965	0.4432
pH	−0.1900	−0.6551	0.0840	−0.2828	**0.5248**
EC	0.3886	−0.0839	**0.5363**	0.4553	0.4652
Alkalinity	0.0419	−0.8558	−0.1334	−0.1990	0.1804
Be	0.2417	**0.5150**	−0.1384	**0.5436**	0.4214
V	−0.4874	**0.7351**	0.2189	0.0749	0.3380
Cr	−0.2476	**0.8574**	0.2698	−0.0474	−0.1765
Mn	**0.8936**	0.0911	−0.0276	−0.3416	−0.0365
Co	**0.9687**	0.0988	0.0185	−0.1507	0.0416
Ni	**0.9219**	0.1502	0.0891	−0.2885	0.0746
Cu	**0.6030**	0.1119	**0.6812**	−0.1419	−0.1886
Zn	0.4386	0.1176	**0.7799**	−0.0682	0.1081
As	−0.1061	0.7733	−0.2425	−0.4112	0.2689
Se	−0.1487	**0.8540**	−0.2373	−0.3470	0.2594
Cd	0.9591	0.0341	−0.0101	−0.1859	0.0197
Sb	**0.5607**	−0.0521	−0.2696	0.3223	−0.2290
Ba	**0.7120**	−0.0234	−0.2423	0.4370	0.3215
Hg	−0.2335	0.1716	**0.6983**	0.0980	−0.1280
Pb	−0.1928	−0.0659	**0.9167**	0.0710	−0.1803
Eigenvalue	5.7517	4.3560	3.3038	1.5992	1.4443
Variability (%)	30.2721	22.9262	17.3885	8.4166	7.6015
**Cumulative (%)**	**30.2721**	**53.1983**	**70.5868**	**79.0034**	**86.6050**

Bold values show the parameters in a specific factor loading.

**Table 7:** Average chronic daily intake (CDI) values, in mg/kg/day, of geothermal water and boreholes for adults and children within Soutpansberg.

		TSS	TSW	SGS	SGW	MPS	MPW	SAW	SH1	SH2	BH1	BH2	SCC	TTP
Be	Children	2.19 × 10^−1^	7.01 × 10^−1^	1.61 × 10^−1^	1.08 × 10^−3^	3.12 × 10^−1^	6.16 × 10^−1^	6.00 × 10^−3^	3.85 × 10^−1^	4.23 × 10^−1^	5.24 × 10^−1^	8.11 × 10^−1^	6.07 × 10^−1^	1.08 × 10^−3^
	Adult	5.74 × 10^−2^	1.83 × 10^−1^	4.21 × 10^−2^	2.83 × 10^−4^	8.16 × 10^−2^	1.61 × 10^−1^	1.57 × 10^−3^	1.01 × 10^−1^	1.11 × 10^−1^	1.37 × 10^−1^	2.13 × 10^−1^	1.59 × 10^−1^	2.83 × 10^−4^
V	Children	2.20	2.01	1.62	1.75	1.95	1.68	3.85 × 10^−1^	1.62	2.12	6.14 × 10^−1^	1.50	2.14	5.54 × 10^−1^
	Adult	5.77 × 10^−1^	5.26 × 10^−01^	4.25 × 10^−1^	4.59 × 10^−1^	5.12 × 10^−1^	4.39 × 10^−1^	1.01 × 10^−1^	4.26 × 10^−1^	5.54 × 10^−1^	1.61 × 10^−1^	3.92 × 10^−1^	5.60 × 10^−1^	1.45 × 10^−1^
Cr	Children	1.50	1.04	1.26	7.97 × 10^−1^	1.27	1.01	1.08 × 10^−2^	1.25	1.33	8.39 × 10^−1^	7.78 × 10^−1^	1.46	1.08
	Adult	3.92 × 10^−1^	2.71 × 10^−1^	3.29 × 10^−1^	2.09 × 10^−1^	3.32 × 10^−1^	2.64 × 10^−1^	2.83 × 10^−3^	3.27 × 10^−1^	3.48 × 10^−1^	2.20 × 10^−01^	2.04 × 10^−1^	3.82 × 10^−1^	2.84 × 10^−1^
Mn	Children	3.20 × 10^−1^	2.67 × 10^−1^	1.24	3.07	4.39	1.28 × 10^−1^	2.88 × 10^−2^	1.50 × 10^−1^	2.34 × 10^−1^	1.29 × 10^1^	1.99 × 10^−1^	1.83 × 10^−1^	1.76
	Adult	8.38 × 10^−2^	6.99 × 10^−2^	3.24 × 10^−1^	8.03 × 10^−1^	1.15	3.35 × 10^−2^	7.54 × 10^−3^	3.94 × 10^−2^	6.12 × 10^−2^	3.38	5.20 × 10^−2^	4.79 × 10^−2^	4.62 × 10^−1^
Co	Children	2.51 × 10^−2^	3.34 × 10^−2^	5.18 × 10^−2^	4.27 × 10^−2^	3.49 × 10^−2^	4.37 × 10^−2^	4.80 × 10^−3^	2.23 × 10^−2^	3.13 × 10^−2^	4.11 × 10^−1^	6.10 × 10^−2^	2.86 × 10^−2^	2.04 × 10^−2^
	Adult	6.58 × 10^−3^	8.75 × 10^−3^	1.36 × 10^−2^	1.12 × 10^−2^	9.15 × 10^−3^	1.15 × 10^−2^	1.26 × 10^−3^	5.85 × 10^−3^	8.20 × 10^−3^	1.08 × 10^−1^	1.60 × 10^−2^	7.48 × 10^−3^	5.34 × 10^−3^
Ni	Children	2.69 × 10^−1^	3.16 × 10^−1^	1.19 × 10^−1^	1.33 × 10^−1^	2.56 × 10^−1^	1.01 × 10^−1^	8.52 × 10^−2^	9.80 × 10^−2^	1.78 × 10^−1^	1.50	6.54 × 10^−2^	2.26 × 10^−1^	1.89 × 10^−1^
	Adult	7.06 × 10^−2^	8.28 × 10^−2^	3.12 × 10^−2^	3.47 × 10^−2^	6.72 × 10^−2^	2.65 × 10^−2^	2.23 × 10^−2^	2.57 × 10^−2^	4.66 × 10^−2^	3.93 × 10^−1^	1.71 × 10^−2^	5.92 × 10^−2^	4.94 × 10^−2^
Cu	Children	1.44	2.25	3.67	7.70 × 10^−3^	1.54 × 10^−1^	1.08 × 10^−3^	4.20 × 10^−2^	1.08 × 10^−3^	2.21 × 10^−1^	3.77	4.13 × 10^−2^	2.58 × 10^−1^	5.78 × 10^−1^
	Adult	3.76 × 10^−1^	5.89 × 10^−1^	9.61 × 10^−1^	2.02 × 10^−3^	4.02 × 10^−2^	2.83 × 10^−4^	1.10 × 10^−2^	2.83 × 10^−4^	5.78 × 10^−2^	9.87 × 10^−1^	1.08 × 10^−2^	6.76 × 10^−2^	1.51 × 10^−1^
Zn	Children	3.75 × 10^1^	5.58 × 10^1^	3.53 × 10^1^	2.34 × 10^+1^	5.92	2.52	1.14 × 10^−1^	1.08 × 10^−3^	1.08 × 10^−3^	4.21 × 10^1^	1.08 × 10^−3^	1.08 × 10^−3^	5.38
	Adult	9.83	1.46 × 10^1^	9.25	6.12	1.55	6.60 × 10^−1^	2.99 × 10^−2^	2.83 × 10^−4^	2.83 × 10^−4^	1.10 × 10^1^	2.83 × 10^−4^	2.83 × 10^−4^	1.41
As	Children	2.45 × 10^−1^	2.42 × 10^−1^	1.62 × 10^−1^	2.37 × 10^−1^	3.27 × 10^−1^	2.52 × 10^−1^	1.21 × 10^−1^	1.24 × 10^−1^	3.65 × 10^−1^	2.30 × 10^−1^	1.55 × 10^−1^	3.66 × 10^−1^	1.56 × 10^−1^
	Adult	6.42 × 10^−2^	6.33 × 10^−2^	4.25 × 10^−2^	6.20 × 10^−2^	8.55 × 10^−2^	6.59 × 10^−2^	3.17 × 10^−2^	3.24 × 10^−2^	9.56 × 10^−2^	6.03 × 10^−2^	4.07 × 10^−2^	9.59 × 10^−2^	4.09 × 10^−2^
Se	Children	6.99 × 10^−1^	7.42 × 10^−1^	4.63 × 10^−1^	6.89 × 10^−1^	1.20	7.70 × 10^−1^	8.16 × 10^−2^	6.08 × 10^−1^	1.31	7.02 × 10^−1^	4.34 × 10^−1^	1.31	3.90 × 10^−1^
	Adult	1.83 × 10^−1^	1.94 × 10^−1^	1.21 × 10^−1^	1.80 × 10^−1^	3.15 × 10^−1^	2.02 × 10^−1^	2.14 × 10^−2^	1.59 × 10^−1^	3.44 × 10^−1^	1.84 × 10^−1^	1.14 × 10^−1^	3.44 × 10^−1^	1.02 × 10^−1^
Cd	Children	6.76 × 10^−3^	2.94 × 10^−3^	1.60 × 10^−3^	6.46 × 10^−3^	9.20 × 10^−4^	1.65 × 10^−2^	2.40 × 10^−3^	1.08 × 10^−3^	8.40 × 10^−4^	8.71 × 10^−2^	1.08 × 10^−3^	8.40 × 10^−4^	5.40 × 10^−3^
	Adult	1.77 × 10^−3^	7.70 × 10^−4^	4.19 × 10^−4^	1.69 × 10^−3^	2.41 × 10^−4^	4.32 × 10^−3^	6.29 × 10^−4^	2.83 × 10^−4^	2.20 × 10^−4^	2.28 × 10^−2^	2.83 × 10^−4^	2.20 × 10^−4^	1.41 × 10^−3^
Sb	Children	6.36 × 10^−3^	2.64 × 10^−3^	3.76 × 10^−3^	7.06 × 10^−3^	4.28 × 10^−3^	2.07 × 10^−2^	3.60 × 10^−3^	6.00 × 10^−4^	1.80 × 10^−3^	1.46 × 10^−2^	9.48 × 10^−3^	1.08 × 10^−3^	1.14 × 10^−2^
	Adult	1.67 × 10^−3^	6.91 × 10^−4^	9.85 × 10^−4^	1.85 × 10^−3^	1.12 × 10^−3^	5.42 × 10^−3^	9.43 × 10^−4^	1.57 × 10^−4^	4.71 × 10^−4^	3.83 × 10^−3^	2.48 × 10^−3^	2.83 × 10^−4^	2.99 × 10^−3^
Ba	Children	1.84 × 10^−1^	3.17	9.36 × 10^−2^	5.09	1.06	3.48	1.25	4.55 × 10^−2^	8.64 × 10^−1^	8.08	8.64	8.21 × 10^−1^	1.08 × 10^−3^
	Adult	4.83 × 10^−2^	8.29 × 10^−1^	2.45 × 10^−2^	1.33	2.76 × 10^−1^	9.11 × 10^−1^	3.27 × 10^−1^	1.19 × 10^−2^	2.26 × 10^−1^	2.12	2.26	2.15 × 10^−1^	2.83 × 10^−4^
Hg	Children	7.33 × 10^−1^	7.45 × 10^−2^	3.91 × 10^−1^	4.83 × 10^−2^	2.18 × 10^−1^	5.17 × 10^−2^	4.20 × 10^−2^	7.94 × 10^−2^	9.58 × 10^−2^	1.84 × 10^−2^	1.92 × 10^−1^	5.52 × 10^−2^	8.64 × 10^−2^
	Adult	1.92 × 10^−1^	1.95 × 10^−2^	1.02 × 10^−1^	1.27 × 10^−2^	5.71 × 10^−2^	1.35 × 10^−2^	1.10 × 10^−2^	2.08 × 10^−2^	2.51 × 10^−2^	4.81 × 10^−3^	5.03 × 10^−2^	1.45 × 10^−2^	2.26 × 10^−2^
Pb	Children	3.38 × 10^−2^	3.9 × 10^−2^	5.87 × 10^−2^	1.08 × 10^−3^	1.08 × 10^−3^	1.08 × 10^−3^	1.08 × 10^−2^	2.05 × 10^−2^	1.08 × 10^−3^	1.08 × 10^−3^	1.08 × 10^−3^	1.08 × 10^−3^	7.68 × 10^−3^
	Adult	8.86 × 10^−3^	1.02 × 10^−2^	1.54 × 10^−2^	2.83 × 10^−4^	2.83 × 10^−4^	2.83 × 10^−4^	2.83 × 10^−3^	5.37 × 10^−3^	2.83 × 10^−4^	2.83 × 10^−4^	2.83 × 10^−4^	2.83 × 10^−4^	2.01 × 10^−3^

**Table 8:** Carcinogenic risk assessment of Cr, Cd, As and Pb, from geothermal springs and boreholes within Soutpansberg, through the ingestion pathway for adults and children.

	Cr		Cd		As		Pb	
Code	Children	Adults	Children	Adults	Children	Adults	Children	Adults
TSS	2.49 × 10^−1^	5.59 × 10^−3^	4.26 × 10^−2^	9.56 × 10^−4^	1.22	2.71 × 10^−2^	8.22 × 10^−5^	5.84 × 10^−8^
TSW	1.73 × 10^−1^	3.88 × 10^−3^	1.85 × 10^−2^	4.16 × 10^−4^	1.21	1.82 × 10^−2^	9.50 × 10^−5^	6.75 × 10^−8^
SGS	2.10 × 10^−1^	4.70 × 10^−3^	1.01 × 10^−2^	2.26 × 10^−4^	8.12 × 10^−1^	2.66 × 10^−2^	1.43 × 10^−4^	1.01 × 10^−7^
SGW	1.33 × 10^−1^	2.98 × 10^−3^	4.07 × 10^−2^	9.13 × 10^−4^	1.18	3.67 × 10^−2^	2.62 × 10^−6^	1.86 × 10^−9^
MPS	2.11 × 10^−1^	4.75 × 10^−3^	5.80 × 10^−3^	1.30 × 10^−4^	1.63	2.82 × 10^−2^	2.62 × 10^−6^	1.86 × 10^−9^
MPW	1.68 × 10^−1^	3.77 × 10^−3^	1.04 × 10^−1^	2.33 × 10^−3^	1.26	1.36 × 10^−2^	2.62 × 10^−6^	1.86 × 10^−9^
SAW	1.80 × 10^−3^	4.04 × 10^−5^	1.51 × 10^−2^	3.39 × 10^−4^	6.06 × 10^−1^	1.39 × 10^−2^	2.62 × 10^−5^	1.86 × 10^−8^
SH1	2.08 × 10^−1^	4.67 × 10^−3^	6.80 × 10^−3^	1.53 × 10^−4^	6.19 × 10^−1^	4.10 × 10^−2^	4.98 × 10^−5^	3.54 × 10^−8^
SH2	2.22 × 10^−1^	4.97 × 10^−3^	5.29 × 10^−3^	1.19 × 10^−4^	1.83	2.59 × 10^−2^	2.62 × 10^−6^	1.86 × 10^−9^
BH1	1.40 × 10^−1^	3.14 × 10^−3^	5.49 × 10^−1^	1.23 × 10^−2^	1.15	1.74 × 10^−2^	2.62 × 10^−6^	1.86 × 10^−9^
BH2	1.30 × 10^−1^	2.91 × 10^−3^	6.80 × 10^−3^	1.53 × 10^−4^	7.76 × 10^−1^	4.11 × 10^−2^	2.62 × 10^−6^	1.86 × 10^−9^
SCC	2.43 × 10^−4^	5.45 × 10^−5^	5.29 × 10^−4^	1.19 × 10^−4^	1.83 × 10^-5^	1.75 × 10^−6^	2.62 × 10^−6^	1.86 × 10^−9^
TTP	1.81 × 10^−4^	4.05 × 10^−5^	3.40 × 10^−5^	7.64 × 10^−4^	7.80 × 10^−4^	3.35 × 10^−4^	1.87 × 10^−5^	1.32 × 10^−8^

## Appendix A

**Table A1:** Hazard quotient for geothermal springs and boreholes within Soutpansberg.

		TSS	TSW	SGS	SGW	MPS	MPW	SAW	SH1	SH2	BH1	BH2	SCC	TTP	HI−Total
Be															
Children	Ingestion	1.10	3.50	8.03 × 10^−1^	5.40 × 10^−3^	1.56	3.08	3.00 × 10^−2^	1.92	2.12	2.62	4.06	3.04	5.40 × 10^−3^	**2.38 × 10^1^**
	Dermal	−	−	−	−	−	−	−	−	−	−	−	−	−	**−**
Adult	Ingestion	2.46 × 10^−2^	7.86 × 10^−2^	1.80 × 10^−2^	1.21 × 10^−4^	3.50 × 10^−2^	6.91 × 10^−2^	6.73 × 10^−4^	4.32 × 10^−2^	4.75 × 10^−2^	5.88 × 10^−2^	9.11 × 10^−2^	6.82 × 10^−2^	1.21 × 10^−4^	**5.35 × 10^−1^**
	Dermal	−	−	−	−	−	−	−	−	−	−	−	−	−	**−**
**V**															
Children	Ingestion	4.37 × 10^−1^	3.98 × 10^−1^	3.22 × 10^−1^	3.47 × 10^−1^	3.88 × 10^−1^	3.32 × 10^−1^	7.64 × 10^−2^	3.22 × 10^−1^	4.20 × 10^−1^	1.22 × 10^−1^	2.97 × 10^−1^	4.25 × 10^−1^	1.10 × 10^−1^	**4.00**
	Dermal	−	−	−	−	−	−	−	−	−	−	−	−	−	**−**
Adult	Ingestion	9.81 × 10^−3^	8.94 × 10^−3^	7.22 × 10^−3^	7.80 × 10^−3^	8.70 × 10^−3^	7.46 × 10^−3^	1.72 × 10^−3^	7.24 × 10^−3^	9.42 × 10^−3^	2.74 × 10^−3^	6.66 × 10^−3^	9.53 × 10^−3^	2.47	**8.97 × 10^−2^**
	Dermal	−	−	−	−	−	−	−	−	−	−	−	−	−	**−**
**Cr**															
Children	Ingestion	4.98 × 10^−1^	3.46 × 10^−1^	4.19 × 10^−1^	2.66 × 10^−1^	4.23 × 10^−1^	3.36 × 10^−1^	3.60 × 10^−3^	4.16 × 10^−1^	4.43 × 10^−1^	2.80 × 10^−1^	2.59 × 10^−1^	4.86 × 10^−1^	3.61 × 10^−1^	**4.54**
	Dermal	1.83 × 10^−3^	1.27 × 10^−3^	1.54 × 10^−3^	9.74 × 10^−4^	1.55 × 10^−3^	1.23 × 10^−3^	1.32 × 10^−5^	1.53 × 10^−3^	1.63 × 10^−3^	1.03 × 10^−3^	9.51 × 10^−4^	1.78 × 10^−3^	1.32 × 10^−3^	**1.66 × 10^−2^**
Adult	Ingestion	1.12 × 10^−2^	7.76 × 10^−3^	9.41 × 10^−3^	5.97 × 10^−3^	9.49 × 10^−3^	7.55 × 10^−3^	8.08 × 10^−5^	9.34 × 10^−3^	9.95 × 10^−3^	6.28 × 10^−3^	5.82 × 10^−3^	1.09 × 10^−2^	8.10 × 10^−3^	**1.02 × 10^−1^**
	Dermal	5.31 × 10^−5^	3.68 × 10^−5^	4.47 × 10^−5^	2.83 × 10^−5^	4.50 × 10^−5^	3.58 × 10^−5^	3.84 × 10^−7^	4.43 × 10^−5^	4.72 × 10^−5^	2.98 × 10^−5^	2.76 × 10^−5^	5.17 × 10^−5^	3.85 × 10^−5^	**4.83 × 10^−4^**
**Mn**															
Children	Ingestion	1.33 × 10^−2^	1.11 × 10^−2^	5.15 × 10^−2^	1.28 × 10^−1^	1.83 × 10^−1^	5.32 × 10^−3^	1.20 × 10^−3^	6.27 × 10^−3^	9.73 × 10^−3^	5.38 × 10^−1^	8.28 × 10^−3^	7.62 × 10^−3^	7.35 × 10^−2^	**1.04**
	Dermal	8.21 × 10^−4^	6.84 × 10^−4^	3.17 × 10^−3^	7.86 × 10^−3^	1.13 × 10^−2^	3.28 × 10^−4^	7.38 × 10^−5^	3.86 × 10^−4^	5.99 × 10^−4^	3.31 × 10^−2^	5.09 × 10^−4^	4.69 × 10^−4^	4.52 × 10^−3^	**6.38 × 10^−2^**
Adult	Ingestion	2.99 × 10^−4^	2.50 × 10^−4^	1.16 × 10^−3^	2.87 × 10^−3^	4.11 × 10^−3^	1.19 × 10^−4^	2.69 × 10^−5^	1.41 × 10^−4^	2.18 × 10^−4^	1.21 × 10^−2^	1.86 × 10^−4^	1.71 × 10^−4^	1.65 × 10^−3^	**2.33 × 10^−2^**
	Dermal	2.38 × 10^−5^	1.99 × 10^−5^	9.21 × 10^−5^	2.28 × 10^−4^	3.27 × 10^−4^	9.52 × 10^−6^	2.15 × 10^−6^	1.12 × 10^−5^	1.74 × 10^−5^	9.61 × 10^−4^	1.48 × 10^−5^	1.36 × 10^−5^	1.31 × 10^−4^	**1.85 × 10^−3^**
**Co**															
Children	Ingestion	1.26 × 10^−3^	1.67 × 10^−3^	2.59 × 10^−3^	2.13 × 10^−3^	1.75 × 10^−3^	2.19 × 10^−3^	2.40 × 10^−4^	1.12 × 10^−3^	1.57 × 10^−3^	2.05 × 10^−2^	3.05 × 10^−3^	1.43 × 10^−3^	1.02 × 10^−3^	**4.05 × 10^−2^**
	Dermal	1.62 × 10^−2^	2.15 × 10^−2^	3.33 × 10^−2^	2.75 × 10^−2^	2.25 × 10^−2^	2.81 × 10^−2^	3.09 × 10^−3^	1.44 × 10^−2^	2.01 × 10^−2^	2.64 × 10^−1^	3.92 × 10^−2^	1.84 × 10^−2^	1.31 × 10^−2^	**5.21 × 10^−1^**
Adult	Ingestion	2.82 × 10^−5^	3.75 × 10^−5^	5.82 × 10^−5^	4.79 × 10^−5^	3.92 × 10^−5^	4.91 × 10^−5^	5.39 × 10^−6^	2.51 × 10^−5^	3.52 × 10^−5^	4.61 × 10^−4^	6.84 × 10^−5^	3.21 × 10^−5^	2.29 × 10^−5^	**9.10 × 10^−4^**
	Dermal	4.69 × 10^−4^	6.25 × 10^−4^	9.69 × 10^−4^	7.98 × 10^−4^	6.53 × 10^−4^	8.17 × 10^−4^	8.97 × 10^−5^	4.17 × 10^−4^	5.85 × 10^−4^	7.67 × 10^−3^	1.14 × 10^−3^	5.34 × 10^−4^	3.81 × 10^−4^	**1.52 × 10^−2^**
**Ni**															
Children	Ingestion	1.35 × 10^−2^	1.58 × 10^−2^	5.95 × 10^−3^	6.63 × 10^−3^	1.28 × 10^−2^	5.06 × 10^−3^	4.26 × 10^−3^	4.90 × 10^−3^	8.89 × 10^−3^	7.51 × 10^−2^	3.27 × 10^−3^	1.13 × 10^−2^	9.43 × 10^−3^	**1.77 × 10^−1^**
	Dermal	1.08 × 10^−8^	1.26 × 10^−8^	4.75 × 10^−9^	5.30 × 10^−9^	1.02 × 10^−8^	4.04 × 10^−9^	3.40 × 10^−9^	3.92 × 10^−9^	7.11 × 10^−9^	6.00 × 10^−8^	2.61 × 10^−9^	9.03 × 10^−9^	7.53 × 10^−9^	**1.41 × 10^−7^**
Adult	Ingestion	3.02 × 10^−4^	3.55 × 10^−4^	1.34 × 10^−4^	1.49 × 10^−4^	2.88 × 10^−4^	1.13 × 10^−4^	9.56 × 10^−5^	1.10 × 10^−4^	2.00 × 10^−4^	1.69 × 10^−3^	7.34 × 10^−5^	2.54 × 10^−4^	2.12 × 10^−4^	**3.97 × 10^−3^**
	Dermal	5.13 × 10^−6^	6.02 × 10^−6^	2.26 × 10^−6^	2.52 × 10^−6^	4.88 × 10^−6^	1.92 × 10^−6^	1.62 × 10^−6^	1.87 × 10^−6^	3.38 × 10^−6^	2.86 × 10^−5^	1.24 × 10^−6^	4.30 × 10^−6^	3.59 × 10^−6^	**6.73 × 10^−5^**
**Cu**															
Children	Ingestion	3.88 × 10^−2^	6.08 × 10^−2^	9.92 × 10^−2^	2.08 × 10^−4^	4.15 × 10^−3^	2.92 × 10^−5^	1.14 × 10^−3^	2.92 × 10^−5^	5.96 × 10^−3^	1.02 × 10^−1^	1.12 × 10^−3^	6.98 × 10^−3^	1.56 × 10^−2^	**3.36 × 10^−1^**
	Dermal	2.19 × 10^−4^	3.44 × 10^−4^	5.61 × 10^−4^	1.18 × 10^−6^	2.35 × 10^−5^	1.65 × 10^−7^	6.42 × 10^−6^	1.65 × 10^−7^	3.37 × 10^−5^	5.75 × 10^−4^	6.31 × 10^−6^	3.94 × 10^−5^	8.83 × 10^−5^	**1.90 × 10^−3^**
Adult	Ingestion	8.71 × 10^−4^	1.37 × 10^−3^	2.23 × 10^−3^	4.67 × 10^−6^	9.32 × 10^−5^	6.55 × 10^−7^	2.55 × 10^−5^	6.55 × 10^−7^	1.34 × 10^−4^	2.29 × 10^−3^	2.50 × 10^−5^	1.57 × 10^−4^	3.51 × 10^−4^	**7.54 × 10^−3^**
	Dermal	6.37 × 10^−6^	9.99 × 10^−6^	1.63 × 10^−5^	3.42 × 10^−8^	6.82 × 10^−7^	4.79 × 10^−9^	1.86 × 10^−7^	4.79 × 10^−9^	9.80 × 10^−7^	1.67 × 10^−5^	1.83 × 10^−7^	1.15 × 10^−6^	2.57 × 10^−6^	**5.52 × 10^−5^**
**Zn**															
Children	Ingestion	1.25 × 10^−1^	1.86 × 10^−1^	1.18 × 10^−1^	7.78 × 10^−2^	1.97 × 10^−2^	8.40 × 10^−3^	3.80 × 10^−4^	3.60 × 10^−6^	3.6 × 10^-6^	1.40 × 10^−1^	3.60 × 10^−6^	3.60 × 10^−6^	1.79 × 10^−2^	**6.94 × 10^−1^**
	Dermal	1.84 × 10^−3^	2.73 × 10^−3^	1.73 × 10^−3^	1.14 × 10^−3^	2.90 × 10^−4^	1.23 × 10^−4^	5.57 × 10^−6^	5.28 × 10^−8^	5.28 × 10^−8^	2.06 × 10^−3^	5.28 × 10^−8^	5.28 × 10^−8^	2.63 × 10^−4^	**1.02 × 10^−2^**
Adult	Ingestion	7.30 × 10^−4^	2.34 × 10^−3^	5.35 × 10^−4^	3.60 × 10^−6^	1.04 × 10^−3^	2.05 × 10^−3^	2.00 × 10^−5^	1.28 × 10^−3^	1.41 × 10^−3^	1.75 × 10^−3^	2.70 × 10^−3^	2.02 × 10^−3^	3.60 × 10^−6^	**1.59 × 10^−2^**
	Dermal	5.33 × 10^−5^	7.92 × 10^−5^	5.02 × 10^−5^	3.32 × 10^−5^	8.41 × 10^−6^	3.58 × 10^−6^	1.62 × 10^−7^	1.53 × 10^−9^	1.53 × 10^−9^	5.98 × 10^−5^	1.53 × 10^−9^	1.53 × 10^−9^	7.65 × 10^−6^	**2.96 × 10^−4^**
**As**															
Children	Ingestion	8.17 × 10^−1^	8.05 × 10^−1^	5.41 × 10^−1^	7.89 × 10^−1^	1.09	8.39 × 10^−1^	4.04 × 10^−1^	4.13 × 10^−1^	1.22	7.68 × 10^−1^	5.18 × 10^−1^	1.22	5.20 × 10^−1^	**9.94**
	Dermal	2.99 × 10^−3^	2.95 × 10^−3^	1.98 × 10^−3^	2.89 × 10^−3^	3.99 × 10^−3^	3.07 × 10^−3^	1.48 × 10^−3^	1.51 × 10^−3^	4.46 × 10^−3^	2.82 × 10^−3^	1.90 × 10^−3^	4.47 × 10^−3^	1.91 × 10^−3^	**3.64 × 10^−2^**
Adult	Ingestion	1.83 × 10^−2^	1.81 × 10^−2^	1.21 × 10^−2^	1.77 × 10^−2^	2.44 × 10^−2^	1.88 × 10^−2^	9.07 × 10^−3^	9.27 × 10^−3^	2.73 × 10^−2^	1.72 × 10^−2^	1.16 × 10^−2^	2.74 × 10^−2^	1.17 × 10^−2^	**2.23 × 10^−1^**
	Dermal	8.70 × 10^−5^	8.58 × 10^−5^	5.77 × 10^−5^	8.41 × 10^−5^	1.16 × 10^−4^	8.93 × 10^−5^	4.30 × 10^−5^	4.40 × 10^−5^	1.30 × 10^−4^	8.18 × 10^−5^	5.51 × 10^−5^	1.30 × 10^−4^	5.54 × 10^−5^	**1.06 × 10^−3^**
**Se**															
Children	Ingestion	1.40 × 10^−1^	1.48 × 10^−1^	9.25 × 10^−2^	1.38 × 10^−1^	2.41 × 10^−1^	1.54 × 10^−1^	1.63 × 10^−2^	1.22 × 10^−1^	2.63 × 10^−1^	1.40 × 10^−1^	8.68 × 10^−2^	2.63 × 10^−1^	7.79 × 10^−2^	**1.88**
	Dermal	−	−	−	−	−	−	−	−	−	−	−	−	−	**−**
−	Ingestion	3.14 × 10^−3^	3.33 × 10^−3^	2.08 × 10^−3^	3.09 × 10^−3^	5.40 × 10^−3^	3.46 × 10^−3^	3.66 × 10^−4^	2.73 × 10^−3^	5.90 × 10^−3^	3.15 × 10^−3^	1.95 × 10^−3^	5.89 × 10^−3^	1.75 × 10^−3^	**4.22 × 10^−2^**
	Dermal	−	−	−	−	−	−	−	−	−	−	−	−	−	**−**
**Cd**															
Children	Ingestion	6.7 × 10^−3^	2.94 × 10^−3^	1.60 × 10^−3^	6.46 × 10^−3^	9.20 × 10^−4^	1.65 × 10^−2^	2.40 × 10^−3^	1.08 × 10^−3^	8.40 × 10^−4^	8.71 × 10^−2^	1.08 × 10^−3^	8.40 × 10^−4^	5.40 × 10^−3^	**1.34 × 10^−1^**
	Dermal	2.48 × 10^−5^	1.08 × 10^−5^	5.87 × 10^−6^	2.37 × 10^−5^	3.37 × 10^−6^	6.05 × 10^−5^	8.80 × 10^−6^	3.96 × 10^−6^	3.08 × 10^−6^	3.19 × 10^−4^	3.96 × 10^−6^	3.08 × 10^−6^	1.98 × 10^−5^	**4.91 × 10^−4^**
Adult	Ingestion	1.52 × 10^−4^	6.60 × 10^−5^	3.59 × 10^−5^	1.45 × 10^−4^	2.07 × 10^−5^	3.70 × 10^−4^	5.39 × 10^−5^	2.42 × 10^−5^	1.89 × 10^−5^	1.96 × 10^−3^	2.42 × 10^−5^	1.89 × 10^−5^	1.21 × 10^−4^	**3.01 × 10^−3^**
	Dermal	7.20 × 10^−7^	3.13 × 10^−7^	1.70 × 10^−7^	6.88 × 10^−7^	9.80 × 10^−8^	1.76 × 10^−6^	2.56 × 10^−7^	1.15 × 10^−7^	8.95 × 10^−8^	9.28 × 10^−6^	1.15 × 10^−7^	8.95 × 10^−8^	5.75 × 10^−7^	**1.43 × 10^−5^**
**Sb**															
Children	Ingestion	1.59 × 10^−2^	6.60 × 10^−3^	9.40 × 10^−3^	1.76 × 10^−2^	1.07 × 10^−2^	5.18 × 10^−2^	9.00 × 10^−3^	1.50 × 10^−3^	4.50 × 10^−3^	3.66 × 10^−2^	2.37 × 10^−2^	2.70 × 10^−3^	2.85 × 10^−2^	**2.18 × 10^−1^**
	Dermal	−	−	−	−	−	−	−	−	−	−	−	−	−	**−**
Adult	Ingestion	3.57 × 10^−4^	1.48 × 10^−4^	2.11 × 10^−4^	3.96 × 10^−4^	2.40 × 10^−4^	1.16 × 10^−3^	2.02 × 10^−4^	3.37 × 10^−5^	1.01 × 10^−4^	8.22 × 10^−4^	5.32 × 10^−4^	6.06 × 10^−5^	6.40 × 10^−4^	**4.90 × 10^−3^**
	Dermal	−	−	−	−	−	−	−	−	−	−	−	−	−	**−**
**Ba**															
Children	Ingestion	9.22 × 10^−4^	1.58 × 10^−2^	4.68 × 10^−4^	2.54 × 10^−2^	5.28 × 10^−3^	1.74 × 10^−2^	6.25 × 10^−3^	2.27 × 10^−4^	4.32 × 10^−3^	4.04 × 10^−2^	4.32 × 10^−2^	4.10 × 10^−3^	5.40 × 10^−6^	**1.64 × 10^−1^**
	Dermal	−	−	−	−	−	−	−	−	−	−	−	−	−	**−**
Adult	Ingestion	2.07 × 10^−5^	3.55 × 10^−4^	1.05 × 10^−5^	5.71 × 10^−4^	1.18 × 10^−4^	3.91 × 10^−4^	1.40 × 10^−4^	5.10 × 10^−6^	9.70 × 10^−5^	9.07 × 10^−4^	9.70 × 10^−4^	9.21 × 10^−5^	1.21 × 10^−7^	**3.68 × 10^−3^**
	Dermal	−	−	−	−	−	−	−	−	−	−	−	−	−	**−**
**Hg**															
Children	Ingestion	2.44	2.48 × 10^−1^	1.30	1.61 × 10^−1^	7.27 × 10^−1^	1.72 × 10^−1^	1.40 × 10^−1^	2.65 × 10^−1^	3.19 × 10^−1^	6.12 × 10^−2^	6.40 × 10^−1^	1.84 × 10^−1^	2.88 × 10^−1^	**6.95**
	Dermal	8.96 × 10^−3^	9.11 × 10^−4^	4.78 × 10^−3^	5.90 × 10^−4^	2.67 × 10^−3^	6.31 × 10^−4^	5.13 × 10^−4^	9.71 × 10^−4^	1.17 × 10^−3^	2.24 × 10^−4^	2.35 × 10^−3^	6.75 × 10^−4^	1.06 × 10^−3^	**2.55 × 10^−2^**
Adult	Ingestion	5.49 × 10^−2^	5.58 × 10^−3^	2.93 × 10^−2^	3.62 × 10^−3^	1.63 × 10^−2^	3.87 × 10^−3^	3.14 × 10^−3^	5.94 × 10^−3^	7.17 × 10^−3^	1.37 × 10^−3^	1.44 × 10^−2^	4.13 × 10^−3^	6.47 × 10^−3^	**1.56 × 10^−1^**
	Dermal	2.60 × 10^−4^	2.65 × 10^−5^	1.39 × 10^−4^	1.72 × 10^−5^	7.74 × 10^−5^	1.83 × 10^−5^	1.49 × 10^−5^	2.82 × 10^−5^	3.40 × 10^−5^	6.52 × 10^−6^	6.81 × 10^−5^	1.96 × 10^−5^	3.07 × 10^−5^	**7.41 × 10^−4^**
**Pb**															
Children	Ingestion	9.67 × 10^−3^	1.12 × 10^−2^	1.68 × 10^−2^	3.09 × 10^−4^	3.09 × 10^−4^	3.09 × 10^−4^	3.09 × 10^−3^	5.86 × 10^−03^	3.09 × 10^−4^	3.09 × 10^−4^	3.09 × 10^−4^	3.09 × 10^−4^	2.19 × 10^−3^	**5.09 × 10^−2^**
	Dermal	2.36 × 10^−4^	2.73 × 10^−4^	4.10 × 10^−4^	7.54 × 10^−6^	7.54 × 10^−6^	7.54 × 10^−6^	7.54 × 10^−5^	1.43 × 10^−04^	7.54 × 10^−6^	7.54 × 10^−6^	7.54 × 10^−6^	7.54 × 10^−6^	5.36 × 10^−5^	**1.24 × 10^−3^**
Adult	Ingestion	2.17 × 10^−4^	2.51 × 10^−4^	3.77 × 10^−4^	6.93 × 10^−6^	6.93 × 10^−6^	6.93 × 10^−6^	6.93 × 10^−5^	1.32 × 10^−04^	6.93 × 10^−6^	6.93 × 10^−6^	6.93 × 10^−6^	6.93 × 10^−6^	4.93 × 10^−5^	**1.14 × 10^−3^**
	Dermal	6.87 × 10^−6^	7.94 × 10^−6^	1.19 × 10^−5^	2.19 × 10^−7^	2.19 × 10^−7^	2.19 × 10^−7^	2.19 × 10^−6^	4.16 × 10^−06^	2.19 × 10^−7^	2.19 × 10^−7^	2.19 × 10^−7^	2.19 × 10^−7^	1.56 × 10^−6^	**3.62 × 10^−5^**

Bold values show the summation of the HQs.
